# Nutritional composition of raw fresh cashew (*Anacardium occidentale* L.) kernels from different origin

**DOI:** 10.1002/fsn3.294

**Published:** 2015-10-06

**Authors:** Ricard Rico, Mònica Bulló, Jordi Salas‐Salvadó

**Affiliations:** ^1^ International Nut and Dried Fruit CouncilReusSpain; ^2^ Department of Biochemistry and BiotechnologyIISPVSchool of MedicineRovira i Virgili UniversityCIBERobn Physiopathology of Obesity and NutritionInstituto de Salud Carlos IIIMadridSpain

**Keywords:** *Anacardium occidentale* L., cashew nut, fatty acids, nutritional composition, nuts, sterols

## Abstract

The total dietary fiber, sugar, protein, lipid profile, sodium, and energy contents of 11 raw cashew kernel (*Anacardium occidentale* L.) samples from India, Brazil, Ivory Coast, Kenya, Mozambique, and Vietnam were determined. Total fat was the major component accounting for 48.3% of the total weight, of which 79.7% were unsaturated FA (fatty acids), 20.1% saturated FA, and 0.2% trans FA. Proteins, with 21.3 g/100 g, were ranked second followed by carbohydrates (20.5 g/100 g). The average sodium content was 144 mg/kg. Fourteen FA were identified among which oleic acid was the most abundant with a contribution of 60.7% to the total fat, followed by linoleic (17.77%), palmitic (10.2%), and stearic (8.93%) acids. The mean energy content was 2525 kJ/100g. Furthermore, the sterol profile and content, amino acids, vitamins, and minerals of four raw cashew kernel samples from Brazil, India, Ivory Coast, and Vietnam were determined. *β*‐Sitosterol with 2380 ± 4 mg/kg fat was the most occurring sterol. Glutamic acid, with 4.60 g/100 g, was the amino acid with highest presence, whereas tryptophan with 0.32 g/100 g was the one with lower presence. Vitamin E with an average contribution of 5.80 mg/100 g was the most abundant vitamin. Potassium with a mean value of 6225 mg/kg was the mineral with highest amount in cashew samples.

## Introduction

Nuts played an important role in diets of many cultures and civilizations for centuries due to its high energy and nutritional value as well as its huge variety of flavors and unique taste. Furthermore, consumption of tree nuts had been linked with several health benefits during the last years due to its particular nutritional composition. Tree nuts are known to contain a high content of unsaturated FA (fatty acids), both mono‐ and polyunsaturated FA, combined with a huge variety of vitamins, minerals, amino acids, phytosterols, and a generous content of fiber. Consumption of nuts incorporated in a healthy diet was associated not only to a reduced risk of cardiovascular disease and mortality (Kris‐Etherton et al. 2008; Ros et al. 2010), especially in case of stroke (Estruch et al. 2013), but also to a decreased risk of metabolic syndrome (Fernández‐Montero et al. 2013; Mitjavila et al. 2013) and diabetes (Kendall et al. 2011). Furthermore, in some studies nuts had been found to improve mental health (Carey et al. 2012; Herbison et al. 2012), increase bone mineral density (Rivas et al. 2013), and decrease the risk of depression (Sanhueza et al. 2013). Its long‐term consumption was also associated with a decreased risk of weight gain and obesity (Bes‐Rastrollo et al. 2009). In addition, no association between nut consumption and weight gain was recently demonstrated in a meta‐analysis of clinical trials (Flores‐Mateo et al. 2013). Furthermore, adding cashew nuts in the diet resulted in an increased antioxidant capacity in subjects with metabolic syndrome (Davis et al. 2007). The benefits of the addition of nuts in a healthy diet in front of a low‐fat diet have been recently highlighted by Estruch et al. (2013) confirming that the incidence of major cardiovascular events and mortality is 30% lower for those individuals consuming a Mediterranean diet supplemented with a handful of nuts a day, compared to those that are advised to consume a low‐fat diet.

Among tree nuts, cashew nuts ranks third in worldwide production (kernel basis), with a world average production of 547,371 metric tons (kernel basis) in the last 10 years with a continuous raising trend. In 2014, the total production of cashew kernels achieved 629,668 MT (metric tons), led by India with 164,286 MT and followed by Vietnam with 119,048 MT, Ivory Coast with 109,583 MT, Guinea‐Bissau with 48,300 MT, and Tanzania with 35,200 MT (unpublished results from the International Nut and Dried Council database).

Cashew nuts, *Anacardium occidentale* L., belongs to the Anacardiaceae family and is an evergreen tree native from northeast region of Brazil which expanded spontaneously in South American countries (Asogwa et al. 2008). During the 16th century, it was introduced into India and Africa by Portuguese (Aiyadurai 1966; Asogwa et al. 2008). From India, cashew trees spread all over southeast Asia. Cashew trees can grow from sea level to an altitude of 1000 m (Davis 1999). The tree produces a soft, shiny, and juicy fruit known as cashew apple which bears a single‐seeded nut in its bottom covered with a hard gray shell. Cashew nut plays a massive social aid in many developing countries, where thousands of families live from cashew cultivation. The processing of cashew nuts in shell is difficult and expensive due to the specific characteristics of the shell. African cashew nuts with shell are mostly processed in India and Vietnam. However, year by year, more processing factories are being implemented in Africa helping the local industry and farmers to obtain more benefit and expanding the harvested areas and families living from its cultivation and becoming one of the most important economies in the growing areas.

Although being the third most produced nut worldwide, to date, very little research has been made on cashews. As for its nutritional composition, phenolic lipids (Shobha et al. 1992), saturated and unsaturated FA, tocopherols, squalenes, and phytosterols (Ryan et al. 2006), bioactive compounds such as *β*‐carotene, lutein, zeaxanthin, *α*‐tocopherol, *γ*‐tocopherol, thiamin, stearic acid, oleic acid, and linoleic acid (Trox et al. 2010) were already identified and determined in cashew nuts. Cashew trees are widely spread over tropical areas close to the equator; therefore, the nutritional composition of cashew nuts may vary by origin. The aim of the present study was to determine the nutrient composition of raw fresh cashew (*Anacardium occidentale* L.) kernels and its variability by origin, including cashews from the major world producing areas such as Brazil, India, Vietnam, and East and West Africa.

## Materials and Methods

### Materials

Raw fresh cashew kernels (*Anacardium occidentale* L.) from 11 different origins were used in this study to determine the total dietary fiber, sugar, protein, lipid profile, salt, and energy contents. Six samples proceed from several parts of India, of which two were from Kerala (south west), one from Goa (central west), one from Panruti (south east), one from Andhra Pradesh (central east), and one from Maharashtra (central India). The rest of the samples originated from Brazil, Ivory Coast, Kenya, Mozambique, and Vietnam. Four samples of raw fresh cashew kernels from Brazil, India, Ivory Coast, and Vietnam were used to determine the sterol profile and content, amino acids, vitamins, and heavy metals. One kilogram sample of each origin was obtained, sent to the central laboratory, was protected from light, and then chilled (<21°C). Composition analysis were conducted within 3 months after harvest, and analysis of dietary fiber, sugar, protein, lipid profile, salt, and energy contents were conducted by duplicate.

### Methods

Total dietary fiber was determined by gravimetry. One hundred grams of sample material was digested with different enzymes at 37°C, fat and sugars were removed by solvent extraction and fibers were precipitated and determined gravimetrically with a LOQ (limit of quantification) lower than 0.5 g/100 g (§64 LFGB L 00.00‐18). Sugars were extracted with hot water/methanol (90/10), insoluble matter was precipitated by Carrez reagent, the extract was filtered by a 0.45‐*μ*m filter and sugars were determined by HPLC (high‐performance liquid chromatography) with RID (refractive index detector) (HPLC‐RID; Agilent Technologies, Santa Clara, CA) using the column Merck LiChospher 100 NH2 (Merck, Darmstadt, Germany) and acetonitrile/water (74/26) as eluent. Fat composition of samples was measured after the hydrolysis with 4 mol/L hydrochloric acid. After filtering, the solid matter was washed until neutral and free from chloride and dried (60°C, vacuum). The matter was extracted by petroleum spirit (boiling range 40–60°C), the solvent was evaporated, and the residue was dried (60°C, vacuum). To measure ash content, the sample material was given in a muffle furnace and the organic matter was combusted. The residue was determined gravimetrically (§64 LFGB L 06.00‐4, mod.). Raw protein content was calculated through titrimetry by multiplying the nitrogen (N2) content by 6.25. The sample material was digested in concentrated sulfuric acid at 400°C. All organic bound nitrogen was converted in ammonium sulfate which was extracted in a water steam distillation as ammonia. Ammonia content was determined by a titration with sulfuric acid and protein was then calculated by multiplying the content of nitrogen with factor 6.25 (§64 LFGB L 06.00‐7, mod.). Water content was determined by drying the sample material at 103°C and the residue (dry matter) was determined gravimetrically. Water content was calculated (100 – dry matter) (§64 LFGB L 06.00‐3, mod.). Fatty acid profile was determined by GC (gas chromatography) with FID (flame ionization detector) (GC‐FID CN10703016) (Agilent, Technologies, Santa Clara, CA). The fat yielded by the fat total method was transesterified with methanol–boron trifluoride reagent. The fatty acid methyl esters were analyzed via GC‐FID. The column used was Supelco SP‐2560, 100 m, 0.25 mm i.d., 0.2 *μ*m phase, and hydrogen as carrier gas (Sigma‐Aldrich, St. Louis, MO). The distribution of the peak areas was calculated as the distribution of the mass fraction of the different FA. Isomers were separated according to their molecular weight, number, and position of double bonds as well as configuration of double bounds (*cis*/*trans*) (DGF C‐VI 10a/11a). To determine sodium content, the sample was incinerated at 550°C and the resulting ash was measured via atomic emission spectroscopy (§64 LFGB L 07.00‐56) (Sherwood Scientific, Cambridge, U.K.). The sterol profile and content was determined by saponification, extraction of the unsaponifiable matter and separation of sterols from the unsaponifiable matter by thin‐layer chromatography, analysis of the derivated sterols by capillary gas–liquid column chromatography with FID detector (GC‐FID US00038388)(Agilent Technologies). Vitamin B6 was extracted from the sample in autoclave using acid hydrolysis followed by enzymatic dephosphorylation, reacted with glyoxylic acid in the presence of Fe^2+^, and then reduced with sodium borohydride in alkaline medium, and quantified by reverse‐phase HPLC with fluorometric detection (EX [excitation wavelengths]: 290 nm [nanometers], EM [emission wavelengths]: 395 nm) (EN 14164:2008). Vitamin D3 was saponified using alcoholic potassium hydroxide solution and extracted with hexane:ethylacetate. The extract is concentrated and cleaned up by solid phase extraction (SPE), followed by normal phase semipreparative, and determined by reverse‐phase HPLC with DAD (diode array detection) at 265 nm (Agilent Technologies/Thermo Fisher Scientifics Inc., Waltham, MA) (EN 12821:2009). *β*‐Carotene fat containing samples was released from the sample by hydrolysis using ethanolic potassium hydroxide solution for 16 h at room temperature and extracted one time with ethanol:hexane (4:3 v/v) and two times with hexane. The determination was carried out by reverse‐phase HPLC with ultraviolet (UV)/DAD detection at 450 nm (Agilent Technologies/Thermo Fisher Scientifics Inc.) (EN 12823‐2:2000). Vitamin A (retinol) was released from the sample by alkaline hydrolysis using ethanolic potassium hydroxide solution, extracted three times with hexane:ethylacetate (85:15 v/v), and determined by RP‐HPLC (reverse‐phase HPLC) with UV/DAD detection at 325 nm (Agilent Technologies/Thermo Fisher Scientifics Inc.) (EN 12823‐1:2000). Vitamin B1 was extracted by acid hydrolysis followed by enzyme dephosphorylation and quantified by RP‐HPLC with fluorometric detection (EX:368 nm, EM: 440 nm) after postcolumn oxidation to thiochrome (Agilent Technologies/Thermo Fisher Scientifics Inc.) (EN 14122:2006 mod.). Vitamin B2 (riboflavin) was extracted from the sample using acid hydrolysis and quantified by RP‐HPLC with fluorometric detection (EX: 468 nm, EM: 520 nm) (Agilent Technologies/Thermo Fisher Scientifics Inc.) (En 14152:2006, mod.). Vitamin B5 (pantothenic acid) was measured microbiologically comparing *Lactobacillus plantarum* (ATCC 8014) growth response to calibration solutions (AOAC 945.74/45.2.05 [1990]). Vitamin B8 (biotin) was also measured microbiologically with *Lactobacillus plantarum* (ATCC 8014) and compared to calibration solutions (LST AB 266.1, 1995). Vitamin B9 (total folate) was calculated microbiologically with *Lactobacillus rhamnosus* (ATCC 8043) and compared to calibration solutions (AOAC 45.2.09 [2004]). Vitamin B12 was extracted from the sample in autoclave using a buffered solution, measured microbiologically with *Lactobacillus leichmannii* (ATCC 7830) and compared to calibration solutions (AOAC 952.20/45.2.02). Vitamin C (ascorbic acid + dehydroascorbic acid) was measured by extraction in an aqueous solution containing trichloroacetic acid and the antioxidant tris(2‐carboxyethyl)phosphine. Vitamin C is degraded in a basic environment. The final extract was analyzed by reverse‐phase HPLC with UV detection at 265 nm (Agilent Technologies) (Food Chemistry, 94 626–631). Vitamin K1 was enzymatically treated to remove fat and extracted by *n*‐hexane, the determination was carried out using reverse‐phase HPLC with postcolumn reduction and fluorometric detection (EX: 243 nm, EM: 430 nm) (Agilent Technologies/Thermo Fisher Scientifics Inc.) (EN 14148:2003). Vitamin D2 was saponified in the sample using alcoholic potassium hydroxide solution and extracted with hexane:ethylacetate, the extract was concentrated and cleaned up by solid‐phase extraction followed by normal‐phase semipreparative and then determined by RP‐HPLC with DAD at 265 nm (Agilent Technologies/Thermo Fisher Scientifics Inc.) (EN 12821:2009). Vitamin E (tocopherol profile) (includes *δ*‐tocopherol, *α*‐tocopherol, *β*‐tocopherol, and *γ*‐tocopherol) was released from the sample by alkaline hydrolysis using ethanolic potassium hydroxide solution and then extracted three times with hexane:ethylacetate (85:15 v/v); the determination was carried out by reverse‐phase HPLC with FLD detection (EX/EM: 290/327 nm) (Agilent Technologies/Thermo Fisher Scientifics Inc.) (EN 12822:2000). Vitamin B3 (total niacin) was extracted from the sample in a mild hydrochloric solution at 100°C, adjusted to pH 4.5 with sodium acetate and filtrated; the determination was carried out by RP‐HPLC‐FLD (EX: 322 nm, EM: 380 nm) after a postcolumn oxidation with hydrogen peroxide and Cu(II) ions activated by UV radiation at 365 nm (Agilent Technologies/Thermo Fisher Scientifics Inc.) (EN 15652:2009). Amino acid profile was determined with a Biochrom 30 amino acid analyzer (Biochrom Ltd, Cambourne, UK). Heavy metals (Na, K, Mg, P, Ca, Zn, Se, and Fe) were digested with nitric acid using microwave technique at temperatures up to 235°C. For heavy metals, the sample was digested with nitric acid using microwave technique at temperatures up to 235°C and analyzed by atomic absorption spectroscopy (PerkinElmer, Waltham, MA), ICP‐OES (inductively coupled plasma optical emission spectroscopy) (Varian Inc./Agilent Technologies) and ICP‐MS (inductively coupled plasma mass spectroscopy) (Agilent Technologies) (DIN EN ISO 11885, mod.).

## Results and Discussion

### Dietary fiber, sugars, protein, lipids profile, salt, and energy contents

The water content of cashew kernels was 3.8 ± 0.8% fresh weight of edible food (Table 1). Total protein content was 21.3 ± 0.8% (Table 1). Content on sodium had an average of 144 ± 32 mg/kg (Table 1).

**Table 1:** Water, protein, ash, and sodium contents of the different cashew samples

	Central West India—Goa origin	South West India—Kerala origin 1	South West India—Kerala origin 2	South East India—Panruti origin	Central East India—Andhra Pradesh origin	Central India—Maharashtra origin	Ivory Coast	Brazil	Viet Nam	Kenya	Mozambique	Mean	SD
Water content (g/100 g)	3.5	4.0	4.4	2.3	3.6	4.0	3.4	5.3	4.4	3.9	3.4	3.8	0.8
Raw Protein (g/100 g)	20.1	21.5	21.3	23.0	21.6	20.5	21.4	20.2	21.0	21.5	22.0	21.3	0.8
Ash (g/100 g)	2.5	2.6	2.5	2.8	2.5	2.4	2.6	2.6	2.4	2.5	2.6	2.5	0.1
Sodium (Na) (mg/kg)	120	165	175	105	150	140	115	140	105	205	165	144	32

Fat is the major macronutrient present in cashews accounting for 48.3 ± 1.6% (average) of the total weight, of which 61.8 ± 1.8% were monounsaturated FA, 17.9 ± 0.8% polyunsaturated FA, 20.1 ± 1.7% saturated FA, and 0.19 ± 0.02% trans FA (Table 2).

**Table 2:** Fatty acid profile and content, % weight of total fatty acids of the different cashew samples

	Central West India—Goa origin	South West India—Kerala origin 1	South West India—Kerala origin 2	South East India—Panruti origin	Central East India—Andhra Pradesh origin	Central India—Maharashtra origin	Ivory Coast	Brazil	Viet Nam	Kenya	Mozambique	Mean	SD
Total fatty acid (g/100 g)	49.05	48.60	48.20	48.05	49.35	49.40	49.25	47.40	45.05	50.40	46.20	48.27	1.55
*Fatty acid profile, % weight of total FA*													
Saturated fatty acid	20.25	20.55	20.45	21.55	22.30	20.30	16.95	17.30	21.65	19.90	20.00	20.11	1.66
Monounsaturated fatty acid	60.90	61.65	61.50	60.65	60.75	60.20	64.95	65.40	60.85	62.05	60.70	61.78	1.76
Polyunsaturated fatty acid	18.65	17.60	17.85	17.65	16.75	19.30	17.95	17.10	17.25	17.90	19.10	17.92	0.81
Trans fatty acid	0.20	0.20	0.15	0.20	0.20	0.20	0.15	0.20	0.20	0.20	0.20	0.19	0.02
C12:0 Dodecanoic acid (lauric acid)	n/d	n/d	n/d	n/d	0.25	n/d	n/d	n/d	n/d	n/d	n/d	0.02	0.08
C16:0 Hexadecanoic acid (palmitic acid)	9.90	10.65	10.35	10.40	10.65	10.00	8.30	8.65	10.85	10.70	9.80	10.02	0.84
C16:1 Hexadecenoic acid (palmitoleic acid) (C16:1 n7)	0.40	0.40	0.40	0.40	0.40	0.40	0.40	0.40	0.40	0.50	0.40	0.41	0.03
C17:0 Heptadecanoic acid (margaric acid)	0.10	0.10	0.10	0.10	0.10	0.10	0.10	0.10	0.10	0.10	0.10	0.10	0.00
C18:0 Octadecanoic acid (stearic acid)	9.20	8.75	9.00	9.95	10.35	9.15	7.70	7.60	9.45	8.10	8.95	8.93	0.86
C18:1 Octadecenoic (oleic acid, w‐9) (C18:1n9c)	59.80	60.55	60.50	59.60	59.75	59.15	63.80	64.25	59.80	60.85	59.65	60.70	1.72
Elaidic acid (C18:1n9t)	n/d	n/d	n/d	0.10	n/d	n/d	0.05	0.05	0.10	n/d	n/d	0.03	0.04
C18:1 (Vaccenic acid) (C18:1‐11)	0.40	0.40	0.40	0.40	0.40	0.40	0.50	0.40	0.40	0.40	0.40	0.41	0.03
C18:2 Octadecadienoic acid (linoleic acid) (w‐6)	18.50	17.50	17.70	17.50	16.65	19.15	17.80	16.95	17.10	17.70	18.90	17.77	0.79
C18:3 Octadecatrienoic acid (linolenic acid) (w‐3)	0.10	0.10	0.10	0.15	0.10	0.10	0.10	0.15	0.10	0.20	0.20	0.13	0.04
C20:0 Eicosanoic acid (arachidic acid)	0.70	0.60	0.60	0.70	0.70	0.70	0.50	0.55	0.70	0.60	0.60	0.63	0.07
C20:1 Eicosenoic acid (gadoleic acid)	0.20	0.20	0.20	0.20	0.20	0.20	0.20	0.20	0.20	0.20	0.20	0.20	0.00
C22:0 Docosanoic acid (behenic acid)	0.20	0.10	0.10	0.10	0.10	0.10	0.10	0.10	0.20	0.10	0.10	0.12	0.04
C24:0 Tetracosanoic acid (lignoceric acid)	0.20	0.20	0.20	0.20	0.20	0.20	0.20	0.20	0.20	0.20	0.20	0.20	0.00

n/d: Not detected.

Fourteen FA were identified among which oleic acid was the most abundant with a contribution of 60.7 ± 1.7% to the total fat content, followed by linoleic (17.8 ± 0.8%), palmitic (10.2 ± 0.8%), stearic (8.9 ± 0.9%), and with lower amount of arachidic (0.63 ± 0.07%), palmitoleic (0.41 ± 0.03%), vaccenic (0.41 ± 0.03%), gadoleic (0.20 ± 0.00%), lignoceric (0.20 ± 0.00%), linolenic (0.13 ± 0.04%), behenic (0.12 ± 0.04%), margaric (0.10 ± 0.00%), elaidic (0.03 ± 0.04%), and lauric (0.02 ± 0.08%) acids (Table 2).

Table 3 shows the total fiber, sugar profile, carbohydrates, and energy contents of the different cashew samples analyzed. Among sugars, sucrose was found with a mean of 6.3 ± 0.5 g/100 g. Total dietary fiber had an average content of 3.6 ± 0.2 g/100 g.

**Table 3:** Total energy content and carbohydrate composition of the different cashew samples

	Central West India—Goa origin	South West India—Kerala origin 1	South West India—Kerala origin 2	South East India—Panruti origin	Central East India—Andhra Pradesh origin	Central India—Maharashtra origin	Ivory Coast	Brazil	Viet Nam	Kenya	Mozambique	Mean	SD
Total dietary fiber (g/100 g)	3.4	3.2	3.5	3.7	3.4	3.9	3.8	3.6	3.7	3.7	3.7	3.6	0.2
Sucrose (g/100 g)	6.3	6.1	6.0	6.4	5.8	6.0	6.3	7.3	6.8	5.7	6.6	6.3	0.5
Carbohydrate calculated (diff.) (g/100 g)	21.7	20.1	20.2	20.2	19.6	19.9	19.6	20.9	23.6	18.1	22.2	20.5	1.5
Energy (kJ/100 g)	2551	2531	2516	2542	2553	2545	2549	2482	2453	2566	2490	2525	35.8
Energy (kcal/100 g)	615	610	607	613	616	614	615	598	591	619	600	609	8.9

The energy profile showed a mean content of energy of 2525 ± 35.8 kJ/100 g (Table 3).

### Sterol profile and content, amino acids, vitamins, and heavy metals

Table 4 describes the sterol profile and content of different cashew samples. Total sterols in fat were 286 ± 24 mg/100 g of fat. *β*‐Sitosterol was the major component accounting for 83.3 ± 1.5% of total sterols followed by *δ*‐5‐avenasterol with a 7.5 ± 1.1%, campesterol with a 6.2 ± 0.5%, cholesterol with 0.7 ± 0.4%, and *δ*‐5,24‐stigmastadienol with a 0.7 ± 0.3% of total sterols. On the other hand, 24‐methylene‐cholesterol with 0.13 ± 0.1% and *δ*‐7‐campesterol with 0.08 ± 0.15 were the sterols with lower concentration (Table 4).

**Table 4:** Sterol profile of the different cashew samples

	Vietnamese cashew kernels	Indian cashew kernels Kerala origin	Brazilian cashew kernels	Ivory Coast cashew kernels	Mean	SD
Total sterols in fat (mg/kg fat)	3170	2700	2920	2640	2858	241
*% Total sterols*						
Cholesterol	0.40	0.60	0.50	0.40	0.48	0.10
24‐methylene‐cholesterol	<0.1	0.20	0.20	0.10	0.13	0.10
Campesterol	6.20	6.80	6.30	5.60	6.23	0.49
Stigmasterol	0.20	0.30	0.30	<0.1	0.20	0.06
*δ*‐7‐campesterol	<0.1	0.30	<0.1	<0.1	0.08	0.15
*δ*‐5,23‐stigmastadienol	1.20	<0.1	0.30	0.30	0.45	0.52
Chlerosterol	0.30	0.40	1.10	1.10	0.73	0.43
*β*‐Sitosterol	83.90	82.30	81.80	85.10	83.28	1.51
Sitostanol	0.50	0.30	0.40	0.50	0.43	0.10
*δ*‐5‐avenasterol	6.50	8.60	8.30	6.70	7.53	1.08
*δ*‐5,24‐stigmastadienol	0.70	0.90	0.90	0.30	0.70	0.28

[Correction added on 15 December 2015, after first online publication: the word “Cholesterol” has been revised to “Chlerosterol” in Table 4, column 1, row 9.]

Glutamic acid, with 4.60 ± 0.23 g/100 g, was the amino acid with the highest presence in all the samples, followed by arginine (2.22 ± 0.13 g/100 g), aspartic acid (1.89 ± 0.05 g/100 g), leucine (1.47 ± 0.09 g/100 g), and valine (1.12 ± 0.05 g/100 g). Cysteine + Cystine with 0.40 ± 0.02 g/100 g, methionine with 0.37 ± 0.01 g/100 g, and tryptophan with 0.31 ± 0.02 g/100 g were the amino acids with lower presence (Table 5).

**Table 5:** Amino acid profile of cashews of different origin

	Vietnamese cashew kernels	Indian cashew kernels Kerala origin	Brazilian cashew kernels	Ivory Coast cashew kernels	Mean	SD
*Amino acids g/100 g*						
Alanine	0.812	0.792	0.846	0.810	0.815	0.023
Aspartic acid	1.900	1.850	1.960	1.860	1.893	0.050
Arginine	2.240	2.030	2.340	2.250	2.215	0.131
Cysteine + Cystine	0.396	0.381	0.394	0.429	0.400	0.020
Glutamic acid	4.580	4.320	4.890	4.600	4.598	0.233
Glycine	0.902	0.862	0.902	0.897	0.891	0.019
Histidine	0.475	0.454	0.471	0.471	0.468	0.009
Isoleucine	0.781	0.759	0.828	0.820	0.797	0.033
Leucine	1.440	1.390	1.590	1.470	1.473	0.085
Lysine	0.974	0.924	1.000	0.984	0.971	0.033
Methionine	0.377	0.352	0.367	0.381	0.369	0.013
Phenylalanine	0.916	0.880	0.990	0.945	0.933	0.047
Proline	0.767	0.715	0.753	0.759	0.749	0.023
Serine	1.090	1.050	1.190	1.090	1.105	0.060
Tryptophan (total)	0.307	0.300	0.322	0.337	0.317	0.016
Tyrosine	0.616	0.570	0.691	0.638	0.629	0.050
Valine	1.110	1.060	1.170	1.120	1.115	0.045
Threonine	0.704	0.701	0.813	0.729	0.737	0.052

With an average of 5.80 ± 1.0 mg/100 g, vitamin E (sum of tocopherols) was the most found vitamin in cashew samples. Vitamin B3 (total niacin) was the second one with 1.31 ± 0.19 mg/100 g and vitamin B5 the third with 0.77 ± 0.28 mg/100 g. Provitamin A with 7.63 ± 2.50 *μ*g/100 g and vitamin B12 with 0.06 ± 0.04 *μ*g/100 g were the vitamins with lowest amount found in the samples (Table 6).

**Table 6:** Vitamin profile of cashews of different origin

	Unit	Vietnamese cashew kernels	Indian cashew kernels Kerala origin	Brazilian cashew kernels	Ivory Coast cashew kernels	Mean	SD
Vitamin B1	mg/100 g	0.426	0.487	0.369	0.624	0.477	0.110
Vitamin B12	*μ*g/100 g	0.073	0.038	0.111	0.025	0.062	0.039
Vitamin C (ascorbic acid + dehydroascorbic acid)	mg/100 g	<5 (LOQ)	0.500	<0.5	<0.5	0.125	0.250
Vitamin B2—riboflavin	mg/100 g	0.029	0.030	0.020	0.034	0.028	0.006
Vitamin B5—pantothenic acid, microbiological (mg/100 g)	mg/100 g	0.519	0.577	0.872	1.120	0.772	0.279
Vitamin B8—biotin, microbiological	*μ*g/100 g	61.100	34.100	16.300	22.900	33.600	19.751
Vitamin B9—total folate, microbiological	*μ*g/100 g	35.400	33.400	42.500	45.200	39.125	5.626
*Vitamin E (tocopherol profile)*							
Vitamin E (*α*‐tocopherol)	mg/100 g	0.850	0.289	0.384	0.289	0.453	0.268
Vitamin E (*γ*‐tocopherol)	mg/100 g	3.720	6.210	5.510	4.840	5.070	1.060
*δ*‐Tocopherol	mg/100 g	0.572	0.701	<0.5	<0.5	0.318	0.371
Sum of tocopherols	mg/100 g	4.970	7.200	5.890	5.130	5.798	1.017
Vitamin B6	mg/100 g	0.389	0.414	0.255	0.511	0.392	0.106
Vitamin B3 (total niacin)	mg/100 g	1.180	1.140	1.400	1.530	1.313	0.185
*β*‐Carotene fat containing samples provitamin A	*μ*g/100 g	5.350	5.590	9.950	9.630	7.630	2.499
Vitamin K1	*μ*g/100 g	5.350	16.500	25.000	14.200	15.263	8.077

Potassium with a mean value of 622 ± 59 mg/100 g was the most abundant mineral present in cashew samples, followed by phosphorus with 503 ± 50 mg/100 g, magnesium with 249 ± 12 mg/100 g, and calcium with 41 ± 10 mg/100 g. Sodium with 10.0 ± 3.2 mg/100 g, iron with 5.7 ± 1.1 mg/100 g, zinc with 5.3 ± 0.5 mg/100 g, and selenium with 0.039 ± 0.045 mg/100 g were the heavy metals with lower content (Table 7).

**Table 7:** Mineral profile in cashew of different origins

	Vietnamese cashew kernels	Indian cashew kernels Kerala origin	Brazilian cashew kernels	Ivory Coast cashew kernels	Mean	SD
Iron (mg/100 g)	6.1	4.5	5.1	7.1	5.7	1.1
Zinc (mg/100 g)	4.9	5.5	5.0	5.9	5.3	0.5
Sodium (mg/100 g)	8.3	11.0	14.0	6.6	10.0	3.2
Potassium (mg/100 g)	660.0	620.0	540.0	670.0	622.5	59.1
Magnesium (mg/100 g)	240.0	250.0	240.0	265.0	248.8	11.8
Calcium (mg/100 g)	38.0	46.0	28.0	52.0	41.0	10.4
Phosphorus (mg/100 g)	460.0	510.0	470.0	570.0	502.5	49.9
Selenium (mg/100 g)	<0.2	0.08	0.075	<0.2	0.04	0.04

This is the first study analyzing the nutritional profile of cashew kernel samples from all the largest worldwide growing regions. Although we could observe differences among all samples, in general, the variance found between the nutritional compositions of the analyzed samples was not of great significance.

However, some important differences can be found between the nutritional estimates of our samples and those published by other authors in the literature. In 2010, Trox et al. reported a 66.21 ± 7.87 g/100 g mean content of total fat in nine cashew samples from Indonesia which is considerably higher than our findings 48.3 ± 1.6 g/100 g. None of our samples showed more than 50 g/100 g of total fat. In addition, a higher standard deviation is observed on their results in relation to the fat content. On the other hand, Venkatachalam and Sathe (2006) found slightly lower values for total fat content (43.71 ± 1.13 g/100 g) in three samples from US grocery stores, and Ryan et al. (2006) reported 40.4 ± 2.0 g/100 g (*n* = 3) of total fat in cashews from a local health food store in Cork (Ireland) which are the closest values to our findings. Differences related to the origin of the samples, the processes (storage and manipulation), and the methods used for lipid extraction for its gravity determination may explain these differences observed between studies.

Results on fatty acid composition expressed as percent on total FA were very similar to the previous mentioned studies. Ryan et al. (2006) who found 9 g/100 g less total FA than our results, found a mean concentration of 57.2 g/100 g of oleic acid (18:1), 20.8 g/100 g of linoleic acid (18:2), and 8.7 g/100 g of stearic acid (18:0), in front of 60.7 g/100 g, 17.8 g/100 g, and 8.9 g/100 g of total FA in our study. More closely, Venkatachalam and Sathe (2006) found 61.1 g/100 g, 16.9 g/100 g, and 9.3 g/100 g of total FA of the above FA.

Huge discrepancies in the results of the cashew's sterol content were found in the literature. Ryan et al. (2006) reported 1768.0 ± 210.6 mg/g of fat of *β*‐sitosterol compared to our results of 2380 ± 4 mg/kg. The same occurred with the amount of campesterol reported, they found 105.3 ± 16.0 mg/g fat against 178 ± 1 mg/kg fat of our results, and of stigmasterol, 116.7 ± 12.6 mg/g fat in front of 5.72 ± 0.34 mg/kg fat in our study. Large differences were also observed between the results of Ryan et al. (2006) and ours in relation to the tocopherol content. Their results showed 57.2 ± 6.2 mg/g of *γ*‐tocopherol, much higher than the 5.07 ± 1.06 mg/100 g found in our study. In relation to the *α*‐tocopherol content, Ryan et al. (2006) reported 3.6 ± 1.4 mg/g fat, an amount also higher than in our study, 0.453 ± 0.268 mg/100 g.

The amount of *γ*‐tocopherol (1.10 ± 0.12 mg/100 g) and *α*‐tocopherol (0.29 ± 0.04 mg/100 g) reported by Trox et al. (2010) was similar to that found in our study.

Considerable differences in the amino acid composition were also found from previous studies. In 2007, Adeyeye et al. showed approximately 30% lower amount of amino acids than in our study with the highest differences in proline (0.82 g/100 g crude protein found by Adeyeye et al. 2007, in front of 3.514 g/100 g crude protein found in our study), and threonine contents (1.694 g/100g crude protein found by Adeyeye et al. and 3.459 g/100g crude protein found in our study, respectively). However, slight differences were found in the amino acid profile between the Venkatachalam and Sathe (2006) study and our results.

Among vitamins, the amount of tocopherol content reported by Ryan et al. (2006) was much higher than that found in our study, showing our findings 0.453 mg/100 g of *α*‐tocopherol in front of 360 mg/100 g of oil found by Ryan et al. and 5.07 mg/100 g against 5720 mg/100 g of oil for *γ*‐tocopherol, respectively. Trox et al. (2010) found 0.29 mg/100 g of *α*‐tocopherol and 1.10 mg/100 g of *γ*‐tocopherol. In 2006 Kornsteiner et al. showed 5.1 mg/100g extracted oil for the sum of *β*‐ and *γ*‐tocopherol. Our results on *β*‐carotene were close to the ones found for Trox et al. (2010) (9.57 *μ*g/100 g).

Several differences were also found in mineral composition between our study and others previously published. Compared to our study, Fagbemi (2008) reported in cashew nut flours higher amounts of sodium (19.1 vs. 10.0 mg/100 g), phosphorus (509.8 vs. 502.5 mg/100 g), zinc (8.3 vs. 5.3 mg/100 g), and iron (15.8 vs. 5.7 mg/100 g); and lower amounts of calcium (12.8 vs. 41.0 mg/100 g), potassium (466.8 vs. 622.5 mg/100 g), magnesium (172.2 vs. 248.8 mg/100 g), and selenium (0.0024 vs. 0.04 mg/100 g). On the other hand, compared to the present study, Ijarotimi et al. (2012) reported lower amounts of potassium, magnesium, phosphorus, zinc, and iron and higher amounts of sodium and calcium in cashew nut flour.

The differences observed between studies could be explained from the different methodology used in each study. Nevertheless, most of our results were consistent with the previous published studies. The differences found among samples from different producing regions were not of great significance and could be the result of the soil composition in each area, the climate conditions, and the genetic evolution of the trees in each region (Cheng et al. 2014; Giorgi et al. 2010; Ahmed et al., 2014). Therefore, the nutritional composition of raw cashew kernels is very similar to whatever its growth region. Raw cashew kernels shows equilibrate and high nutritional composition, full of healthy fats and considerable amounts of sterols, amino acids, vitamins, and minerals that have been considered to have beneficial effect on health (Wight et al. 2012; Gupta and Prakash 2014; Ras et al. 2014; Agnew‐Blais et al. 2015).

Cashew nuts represent a good source of unsaturated FA, fiber, sterols, vitamins, and amino acids whatever its grown region, suggesting that their intake contribute to the widely known beneficial roles in health of these nutrients.

One of the limitations of our study is that the determination of total dietary fiber, sugars, protein, lipid profile, salt, and energy contents was carried out in 11 cashew samples from different origins by duplicate and the analysis of the sterol profile and amino acid, vitamin, and mineral content were just conducted in only four samples of different origins with only one determination for each analysis, which limits the reproducibility of our results. However, this is the first study where all the determinations were carried with samples from the worldwide most important growing regions, which makes a much better approximation of the real nutrition content of raw cashew kernels.

## Conflict of Interest

Jordi Salas‐Salvadó serves at the INC World Forum for Nutrition Research and Dissemination and has received grant support from the International Nut and Dried Fruit Council. Mònica Bulló has received a grant support for one of her projects from the American Pistachio Growers and Paramount Frams (USA). Ricardo Rico received grant support from the International Nut and Dried Fruit Council.
